# Qualitative evaluation of an intervention to reduce energy poverty

**DOI:** 10.11606/s1518-8787.2019053001212

**Published:** 2019-08-23

**Authors:** Constanza Jacques-Aviñó, José Luis Dvorzak, Marc Marí-Dell’Olmo, Dolors Rodriguez Arjona, Andrés Peralta, Juli Carrere, Joan Benach, Cristina Ramos, Mònica Plana, María José López

**Affiliations:** I Servei d’Avaluació i Mètodes d’Intervenció. Agència de Salut Pública de Barcelona. Barcelona, España; II Servicio de Medicina Preventiva y Salud Pública. Hospital Universitario de Bellvitge.; Hospitalet de Llobregat. Barcelona, España; III Servei de Qualitat i Intervenció Ambiental (SEQUIA). Agència de Salut Pública de Barcelona. Barcelona, España; IV CIBER de Epidemiolgía y Salud Pública, (CIBERESP). Barcelona, España; V Socióloga e investigadora social (autónoma); VI Associació Ecoserveis. Barcelona, España; VII Universidad Pompeu Fabra. GREDS-EMCONET Grupo de Investigación en Desigualdades en Salud - Employment Conditions Network. Departamento de Ciencias Politicas y Sociales. Barcelona, España; VIII Servei de Sistemes d’Informació Sanitària. Agència de Salut Pública de Barcelona. Barcelona, España; IX Asociación Bienestar y Desarrollo. Barcelona, España

**Keywords:** Energy Supply, policies, Social Inequity, Qualitative Research

## Abstract

**OBJECTIVE:**

To evaluate the “ *Energía, la justa* ” program, aimed at reducing energy poverty in the city of Barcelona, from the point of view of the target population and the workers involved in the intervention.

**METHODS:**

A qualitative, descriptive and exploratory pilot study was carried out, with a phenomenological approach. Twelve semi-structured interviews were conducted: to three users, three energy agents who performed interventions in the homes, and six professionals who participated in the program coordination. A thematic content analysis was carried out using *Atlas-ti software* . Interviews were conducted between October 2016 and March 2017.

**RESULTS:**

Trust in a contact person (e.g. social workers) facilitated the participation, although there were difficulties reaching people who had illegal energy supplies, immigrant women or immigrants who subrent properties. Regarding implementation, home visits, energy efficiency advice and the relationship with energy agents were the best assessed aspects. However, not being able to carry out reforms in deteriorated dwellings was considered a limitation. The program also contributed to raise awareness on energy rights, to save on utility bills and to generate tranquility and social support.

**CONCLUSIONS:**

Programs such as this one can promote energy empowerment and improve psychosocial status. However, strategies with a gender and equity perspective should be considered to reach other vulnerable groups.

## INTRODUCTION

Energy poverty (EP) has been defined as not being able to pay for energy services to satisfy essential domestic needs or to allocate an excessive part of the income for the payment of energy bills^1 , 2^ . This situation is associated with a type of residential exclusion characterized by legal and economic problems^3^ . The intermediate determinants of EP are low household income, high prices of energy services and low energy efficiency of homes, all of which are conditioned, in turn, by structural factors such as socio-environmental policies^3 , 4^ .

Scientific literature has shown that EP can have health consequences, such as circulatory diseases, respiratory problems, as well as anxiety and depression^5^ . There is also an association between living with EP and the excess of winter mortality directly related to macroeconomic indicators^6^ . In fact, cold-related mortality is greater in regions with Mediterranean climatic conditions, which have poorer economic indicators than regions of central and northern Europe, with colder climates^7 , 8^ .

Between 2008 and 2014, Spain was the second country, after Greece, with the highest increase in the price of electricity in the European Union, which coincided with a period of strong economic recession in which unemployment increased and wages decreased^1^ . In 2014, it was estimated that 11% of households, that is, around 5.1 million people, were unable to maintain their homes at an adequate temperature in the cold months^1^ . The population most affected by EP comes from low-income households with some unemployed members^9^ . Likewise, the EP has been associated with single-parent, retired families and women who are engaged in domestic tasks^10 , 11^ .

Interventions carried out in other countries aimed at increasing energy efficiency in homes have been shown to improve thermal comfort, increase savings and, at the indirect benefits level, create a greater sense of security and well-being^12^ . Likewise, it has been observed that carrying out reforms at the architectural level can significantly reduce winter mortality^7^ . However, evaluations of interventions on energy poverty are scarce. Therefore, the objective of this study was to know the valuations of an intervention aimed at reducing EP, incorporating both the perspective of the target persons and the workers involved in the program.

## METHODS

A qualitative, descriptive and exploratory pilot study was conducted from a phenomenological perspective^13^ . This research is part of the evaluation of the “Energía, la justa” program, promoted by the Barcelona City Council during the first semester of 2016^14^ . This program aimed to reduce EP in vulnerable populations through intervention in three areas. First, promoting energy efficiency habits to achieve savings; second, optimizing energy services such as discounting invoices through the processing of the social bonus; and third, installing micro-efficiency measures such as energy-saving light bulbs. On the other hand, the program included an employment insertion for long-term unemployed people, who were offered training to be “energy agents” (EA) and perform the intervention in the homes. The pilot study was conducted between October 2016 and March 2017.

The qualitative evaluation was carried out through 12 semi-structured interviews with eight women and four men. The sample consisted of the following key agents: three people who were the recipients of the program, three EA and six people who worked on the coordination of the program, including a territory technique that carried out tasks of liaison between territorial agents, besides selecting the EA; a technical coordinator, responsible for training and advising the EAs; a program coordinator, responsible for the entities of the territory; and three social service social workers, who made contact with the candidate families to participate.

A script of interviews was designed based on the assessment of the key agents on the implementation and coordination of the program, the implementation and impact of the intervention ( Box 1 ). The selection of the interviewees was carried out through a theoretical sampling, stratified by subgroups. The study was approved by the ethical committee of the Hospital del Mar in Barcelona (code 2017/7286/I) and all informants signed an informed consent form.

**Box 1:** Summary of structured interview script and participant code of key agents.

Key agents	Semi-structured interview topics
Target people (P6, P7, P9)	Assessment of contact with the target people and the possibility of participation (e.g., how was it informed about the possibility of participating in this program? In general, how do you evaluate these first moments?) Overall assessment of program implementation, home visits and the actions of the professionals in charge (e.g., has the program been developed as previously said? What do you like the most and the least? Have structural changes been recommended in your home? Which ones? Could you implement them? Assessment of this aspect) Evaluation of the program impact, positive or negative assessment, suggestions and comments (Do you think that this intervention has improved your health in some way? Has it helped you improve your comfort and well-being at home? What impact have you noticed on energy efficiency – heat utilization, savings...?)
Energy agents (P1, P3, P8)	Assessment of coordination and participation (e.g., how do you think coordination is between the different professionals and institutions involved in the program? Do you think it reaches the people who need it most? How could the diagnosis of possible recipients be improved?
Program Coordinators (P2, P4, P5, P10, P11, P12)	Overall assessment of program implementation (e.g., what are the main positive aspects of the program? When implementing the program, what are the main facilitators and obstacles?) Assessment of the potential effects of the program (e.g., what benefits do you think this program brings to its recipients? Can it also have negative effects on the recipients?)

P: interviewed person

The interviews were recorded on audio and, once transcribed, a thematic content analysis was carried out using Atlas-ti software. The most relevant areas were identified according to the perspective of the key agents. Once the categories were identified, they were examined through a comparison process with the rest of the data^15^ . The saturation of the discourse was used as closure criterion. The results were triangulated by different investigators who participated in the study.

## RESULTS

The results are presented according to the perspective of the different key agents.

### Trust in the Initial Contact person and Distrust in Showing Housing Conditions

Regarding the implementation and coordination of the intervention ( Box 2 ), the recipients saw as positive that the initial contact came from the social worker, considered trustworthy. The clarity of the information received by the EA was assessed, as well as the good treatment and the willingness to collaborate in case of any problem or urgency. In fact, the link created with the EA was a facilitator for the program to be carried out.

**Box 2:** Assessment of the start-up and coordination of the intervention on energy poverty according to different key agents.

Key agents	Sub-categories	Verbatims
Users	Trust in the person of contact	P9: “Well, I was offered the trust of being in an official sector office, such as Habitation”
	Good disposition of the well-assessed energy agents	P7: “Yes, they brought us some sheets explaining it to you, in case we had any doubts..., well, their phone for anything, to call them, if someone came to cut our supply or something,..., to urgent calls...” P6: “Both the treatment, the way of speaking, without hurry... I would give them an A”
Energy agent	Frustration due to lack of resources	P1: “In the material there has been a lack of coordination, at least where I was, there was lack of bulbs, which is very bad. Because the person who talks to the user knows how to say “I do not have light bulbs”
	Distrust in certain vulnerable populations	P3: “... there are also many chastened people, who have broken the electric sticks, of course. We went with the vest, with the identification of the City Council,... but, even so, there were... misgivings.” P1: “...barriers are the people who are afraid and do not let you enter their house because they think you’re going to denounce them”
	Gender inequality and illegality in immigrants renting	P3: “Muslim people, if their husband was not there, “I do not sign anything... “,” Well, when your husband is back, call me again and I come back... “... sometimes there are many people that live here and do not speak anything in the language. P1: “...especially the immigration, when the flats are not theirs, because they rented from another person, and because they are afraid that we will enter their house and denounce the renter, because it is re-rented.
Coordinators	Motivation and involvement were key	P2: “... I think that, maybe, all we tried was to inform them from the beginning..., saying ‘we have this challenge in front of us and we can achieve it... among all, we manage to believe it together... also, everything was very new, we were all there to get out, we were getting out and, in the end left”
	Difficulty in contacting populations in an illegal situation	P5: “... people who have supplies illegally, because they needed electricity or gas or water..., because they did not have resources, economic resources, of their own, but resources to solve; helpless people who did not know, because Social Services, because of collapse and lack of resources, they could not solve the situation,... then, these people were often reluctant to let us enter their house“
	Gender difference in the selection of personnel limited equality	P4: “... I also think that another negative aspect is... that is, a construction profile was chosen when I think it was noticed that it was not necessary. I think that it also limited a lot in terms of equality, because, of course, the percentage of men compared to the percentage of women, within the project, was much higher, something like 70 men for 30 women”

P: interviewed person

According to the AE, despite not always having the necessary materials to do the intervention generated frustration. On the other hand, among the contact barriers, there were people who distrusted the program, either because they had bad previous experiences with the energy companies or because they obtained the energy supplies illegally. In the immigrant population, there were cases with language barriers, such as women who did not speak Spanish and who, in addition, depended on the husband’s consent to participate. It was also commented that a part of the population was afraid to participate, especially immigrants who sublet rooms.

According to the coordinators and the EAs, motivation and involvement were key factors, since, although sometimes they had to improvise, the conviction on the part of the agents facilitated their development. They also commented that the program did not manage to contact people who obtained their energy supplies illegally or had no connection with the social services of the neighborhood. On the other hand, it was pointed out that there was a clear gender inequality in the selection of the EA staff, with a male predominance associated with the construction profile of the workforce.

### Empathy as a Facilitating Factor and Structural Problems as a Barrier

Regarding the implementation of the intervention ( Box 3 ), the following of the EAs in the procedures for tariff optimization, home visits and the installation of materials were very well-assessed by the recipients. Training in energy efficiency was also assessed, although one informant commented that they already knew these tips and that the EAs lacked training.

**Box 3:** Assessment of the intervention on energy poverty implementation according to different key agents.

Key agents	Sub-categories	Verbatims
Users	Satisfaction following rate change	P6: “They manage to lower the power, according to the appliances and the things you have at home, which we, particularly, do not accomplish ‘[...] I have felt very well, very comfortable... all the time...Yes, they have done all of them’”
	Perception of scarce formation of energy agents	P9: “...They need a little more training and information, yes, because even I told them some things they did not know [...] last, one of the gentlemen came alone and the poor guy was a little bit, not ashamed, but I do say something, because I need more information to be able to give...“
Energy agent	Beneficial helps, however, do not solve structural problems	P8: “the best option is to make changes in materials, things that we could not do because we did not have it, and we could not improve much; in any case, we did at least bring thermometers and we told them the right humidity and temperature and if they had a stove, at least; we looked at it to have the right temperature and if they had the nightly rate, we can put it at night, which will decrease energy use”
	The lack of duration as an obstacle to the program	P1: “... at the last minute, when you cannot manage according to how much you earn, you only empower it. To me, that of castrating the program like that, I do not think it is right’ [...] ‘one of the negative things about this project is that it stopped after 6 months. Because when a person makes a commercial and tariff change, and you do it in 1 or 2 months, it gives you time to see if your economics have improved or not.”
Coordinators	Visiting people’s homes allow us to know the housing situation	P10: “Well, because you work, ‘ *in situ* ’, in the person’s own home, where you can see and detect the status and conditions, and after establishing a relationship with the users, which is explained more in details...”
	Previous experience of EA facilitated empathy	P2: “The agents and energy informants are first-hand aware, in many cases, of the situations of energy poverty; in this sense, it is considered it can facilitate communication with the citizens served”.
	Bureaucracy in the procedures was an obstacle	P4: “...a barrier, but from ‘now I ask for this paper, now I ask another, and now, suddenly, what was done here now is not done there, now it can only be done by phone...’. They get dizzy and go around a lot... So, this has also been a very important barrier, which has not made the procedures any easier”

According to the EA, actions that favored saving and improvements in housing were implemented. However, in the floors with structural problems, as in the case of air leaks through windows or doors, the program could not obtain improvements due to not having the necessary resources to carry out reforms. The program was considered to be too short, and interventions were not completed. In addition, the lack of time limited the follow up.

According to the coordinators, the home visits served to detect populations with significant personal and economic problems. Considering that the intervention was free and that the changes in the home were optional facilitated its implementation. On the other hand, the fact that the EAs had experienced EP situations facilitated the development of empathy and involvement in the intervention. Both the coordinators and the EAs identified the procedures that had to be carried out to change the tariffs with the energy companies as an obstacle, considered too bureaucratic and delaying the management.

### Empowerment, Savings and Well-being

Regarding the impact of the intervention ( Box 4 ), the recipients commented that the possibility of saving generated a greater sense of tranquility and well-being. They also pointed out that receiving advice was seen as a learning tool, since they managed to change habits and make better use of household appliances. However, some people indicated that they did not believe that the intervention would improve thermal comfort or health status.

**Box 4:** Assessment of the intervention on energy poverty implementation according to different key agents.

Key agents	Sub-categories	Verbatims
Users	Economic impact and more tranquility	P7: “In my case yes... to be able to have some savings, and to have peace of mind... it is the most basic [...] it is something you notice, both economically and in tranquility... of course you notice it’
	Changing habits to greater energy efficiency	P7: “...you ventilate the whole house and... you let the lights on to warm it up. If before we did not open... let’s go, just a little to ‘keep the heat...’ if before it was done, for example, on weekends, the one who uses washing machines twice... now is going to try to use it 3 times, and of course that consumption has decreased, of course we noticed it...”
	Good valuation but without impact on thermal improvement	P9: “apart from the advice, etc., of social assistance and others, when you have a pain in the back, or anything, being warmer will help you, then, in this case, you endure the pain. I cannot be at the ideal or minimum temperature that it should be, because I can not pay for it. “
Energy agent	Knowledge contributed to empowerment	P8: “...especially, those who were younger and were more proactive, really, we gave them empowerment tools. We saw them saying ‘we had this option... I had no idea, I did not know how to decrease power, I could save a lot of money...’ and here I did notice that people were aware that they could do it”
	Not suffering with cuts of supplies increased tranquility	P1: “Especially people who were with warnings of cut supply , it gives them a year of tranquility, that for a year their supply will not be cut...” “... you explain how you can save with energy efficiency, but you also explain consumption habits…”
	Greater social support	P1: “Then I have also seen people who do not feel alone, who see that someone has listened to them, that they are not invisible to society... they say ‘I was remembered... Even this bad situation I am in.’”
Coordinators	Well-assessed knowledge	P12: “Let’s say that learning, too, to look at and read a light payment invoice, because it is also important, because there is no one who understands it. Well, this is fine, I think this is a positive thing... “
	Potential impact on mental health and other diseases	P2: “Aspects of health, although not verifiable with such a short period of time, there is the possibility of experiencing improvements in terms of mental health, as well as in cases of respiratory diseases and others”
	Low impact on changing habits when there is previous precariousness	P11: “...for their own history,... they are people that habits issues had been enough introduced, eh. They were not people who waste away, and, surely, they were people who did not know or could not change things because the structure of the house did not allow them to, and not because of their own habits”.

Both from the perspective of the EAs and the coordinators, it was commented that the intervention fostered knowledge about more efficient energy consumption. This was seen as an empowerment strategy, especially for the youngest ones. According to the EA, making sure there would be no supply cuts reduced the concern and increased welfare. On the other hand, contacts with some people were beneficial from the social support point of view, especially in cases with few support networks.

According to the coordinators, the training of the recipients contributed to saving and to experiencing a greater sense of comfort thanks to having, for example, the light on for a longer time. Also, the program had a possible impact on aspects of mental health such as stress, as well as on respiratory diseases. However, the impact was low when housing conditions were extremely deficient. In addition, due to the same circumstances of economic restriction of some families, the development of strategies to save on energy consumption was carried out in some cases before the intervention.

## DISCUSSION

The main results of the study indicate that changes in the rates of energy supplies, receiving advice on energy efficiency, as well as carrying out the intervention within the homes were highly valued aspects. On the one hand, the program allowed some recipients to reduce their financial expenses, resulting in more tranquility. On the other hand, the fact of carrying out actions *in situ* allowed to know more about people’s life conditions and establish a closer relationship with them.

Regarding its implementation, the people who participated in the program assessed it as a positive municipal initiative and the professional contact as trustworthy. However, the program did not reach some of the most vulnerable populations. On the one hand, those people who were in a legal limbo with the energy companies showed greater distrust to perform any type of intervention in their homes. However, certain groups of immigrants sublet the housing, an illegality situation that generated greater resistance to participate. This social phenomenon, more frequent for immigrants than for Spaniards, shows social inequality related to the job insecurity, the difficulty of signing contracts with homeowners and the existence of a high-priced housing market with high rent prices^16^ . In addition, a sociocultural pattern related to gender inequalities was observed as immigrant women have fewer opportunities to learn the language of the country of destination and are more exposed to social problems^17^ . This situation placed them at a greater disadvantage to understand and participate in this program, highlighting the importance of developing effective strategies to contact socially excluded and difficult-to-reach sub-populations, especially considering the feminization of the EP^10^ .

Regarding the implementation of the program, there was a good perception of the follow up made by the EAs to make the changes in the type of rate hired, which is especially relevant if one considers that one of the barriers was the bureaucratic procedures with the energy companies. In this sense, it has been observed that electricity and gas bills are seen as those that provide less information and less clear regarding the prices of their services, the conditions of supply and the type of contract^1^ . In addition, the lack of understanding about what is paid in an energy bill contributes to the figure of the “vulnerable consumer,” who sees limited ability to make decisions about the management of their economy^20^ .

The fact that many of the EAs themselves had experienced EP facilitated the creation of a link with the target persons. In this way, the program not only provided energy support, but also offered social support to the extent that people noticed that an institution assessed as important was concerned about their living conditions. There is ample literature that shows the impact of having social support on people’s health, which translates into the need to understand energy vulnerability as a complex social system that should not only consider the energy field^8 , 21 , 22^ .

For the different key agents, the program had an economic impact and in the acquisition of knowledge about rights, which was considered a tool for the empowerment of the population. In general, the informants highlighted that saving contributed to the increase of well-being and less feelings of stress related to the non-payment of invoices. These results would be consistent with those found in another study, which showed that an intervention that improves energy efficiency in the home brings positive impact in the users, by experiencing fewer financial difficulties and by feeling healthier^23^ . However, in this program there were no improvements in people with very poor condition homes, since they required structural reforms. All this suggests the need to carry out actions that intervene on social and economic conditions to help alleviate the EP and its consequences.

Among the limitations of the study, it is noteworthy that we did not include a sample that could handle the experiences according to different characteristics of the target persons. However, having selected different agents allowed to provide a global vision of the program. In addition, this pilot study will lead to another qualitative evaluation that will allow us to analyze different types of people targeted by this intervention. Regarding the strengths, it should be noted that this is the first study at the Spanish state level that has made a qualitative assessment of different agents that have participated in a EP program. Thus, the results obtained will contribute to generate evidence on EP and health, an emerging and very relevant field in our context.

In conclusion, the impact of the program was observed in terms of energy efficiency, at the economic level, and also improvements in the psychosocial health. In addition, the program fostered energy empowerment by increasing knowledge and the capacity to act for energy rights. It is worth noting the complexity that surrounds the population that lives in a EP situation, considering both the social determinants of health and the different axes of inequality such as gender, cultural diversity, migratory status and social class, among others. For this reason, this type of programs should be developed within the framework of agreements that are continued over time and associated with structural social policies that affect the multiple factors taking part on housing problems.
