# Supplementary material for: Qualitative evaluation of an intervention to reduce energy poverty
Source: Rev Saude Publica. 2019 Aug 23;53:62. doi: 10.11606/s1518-8787.2019053001212 (PMC6707618; doi:10.11606/s1518-8787.2019053001212)
Supplement: Translation

# Translation: Evaluación cualitativa de una intervención para reducir la pobreza energética

**Authors:** Constanza Jacques-Aviñó, José Luis Dvorzak, Marc Marí-Dell’Olmo, Dolors Rodriguez Arjona, Andrés Peralta, Juli Carrere, Joan Benach, Cristina Ramos, Mònica Plana, María José López

**Affiliations:** I Servei d’Avaluació i Mètodes d’Intervenció. Agència de Salut Pública de Barcelona. Barcelona, España; II Servicio de Medicina Preventiva y Salud Pública. Hospital Universitario de Bellvitge. Hospitalet de Llobregat. Barcelona, España; III Servei de Qualitat i Intervenció Ambiental (SEQUIA). Agència de Salut Pública de Barcelona. Barcelona, España; IV CIBER de Epidemiolgía y Salud Pública, (CIBERESP). Barcelona, España; V Socióloga e investigadora social (autónoma); VI Associació Ecoserveis. Barcelona, España; VII Universidad Pompeu Fabra. GREDS-EMCONET Grupo de Investigación en Desigualdades en Salud - Employment Conditions Network. Departamento de Ciencias Politicas y Sociales. Barcelona, España; VIII Servei de Sistemes d’Informació Sanitària. Agència de Salut Pública de Barcelona. Barcelona, España; IX Asociación Bienestar y Desarrollo. Barcelona, España

**Keywords:** Abastecimiento de Energía, políticas, Inequidad Social, Investigación Cualitativa

## Abstract

**OBJETIVO:**

Conocer las valoraciones del programa “Energía, la justa” orientado a reducir la pobreza energética en la ciudad de Barcelona, desde la perspectiva de las personas destinatarias y de las personas trabajadoras involucradas en la intervención.

**MÉTODOS:**

Estudio piloto realizado con metodología cualitativa, de tipo descriptivo y exploratorio, analizado desde un enfoque fenomenológico. Se realizaron 12 entrevistas semi-estructuradas: a tres personas destinatarias, a tres agentes energéticos que realizaron intervención en los hogares y a seis profesionales que participaron en la coordinación del programa. Se realizó un análisis de contenido temático mediante el *software* Atlas-ti. Las entrevistas se realizaron entre octubre de 2016 y marzo de 2017.

**RESULTADOS:**

La confianza con una persona de contacto (por ejemplo, trabajadoras sociales) facilitó la participación, pero hubo dificultad para acceder a personas que tenían los suministros ilegales, mujeres inmigrantes o inmigrantes que subalquilaban pisos. Respecto a la implementación, las visitas domiciliarias, el asesoramiento en materia de eficiencia energética y la relación con los agentes energéticos, fueron los aspectos mejor valorados. Sin embargo, se consideró como limitación no poder realizar reformas en viviendas muy deterioradas. A nivel de impacto, el programa contribuyó al conocimiento de los derechos energéticos, a ahorrar en las facturas de los suministros y a generar mayor tranquilidad y soporte social.

**CONCLUSIONES:**

Programas como este fomentan el empoderamiento en materia energética y producen mejoras a nivel psicosocial. No obstante, se deberían desarrollar estrategias con perspectiva de género y de equidad para llegar a otros colectivos especialmente vulnerables.

## INTRODUCCIÓN

La pobreza energética (PE) ha sido definida como no poder pagar servicios energéticos para satisfacer necesidades domésticas esenciales o como destinar una parte excesiva de los ingresos para el pago de las facturas de energía^1 , 2^ . Esta situación se asocia a un tipo de exclusión residencial caracterizada por problemas legales y económicos^3^ . Los determinantes intermedios de padecer PE son los bajos ingresos del hogar, los altos precios de los servicios de energía y la baja eficiencia energética de las viviendas, todo ello condicionado, a su vez, por factores estructurales como las políticas socioambientales^3 , 4^ .

La literatura científica ha mostrado que la PE puede tener consecuencias en la salud, como la aparición de enfermedades circulatorias, problemas respiratorios, además de ansiedad y depresión^5^ . También, existe una asociación entre vivir con PE y el exceso de mortalidad invernal directamente relacionado con indicadores macroeconómicos^6^ . De hecho, la mortalidad relacionada con el frío es mayor en aquellas regiones con condiciones climáticas mediterráneas, que tienen peores indicadores económicos que las regiones de Europa central y del norte, con climas más fríos^7 , 8^ .

Durante los años 2008–2014, España fue el segundo país después de Grecia con el mayor incremento del precio de la electricidad de la Unión Europea, lo que coincidió con un período de fuerte recesión económica en el que aumentó el desempleo y hubo una caída de los salarios^1^ . En el año 2014 se estimó que el 11% de los hogares, es decir unos 5,1 millones de personas, no lograron mantener su vivienda a una temperatura adecuada en los meses fríos^1^ . La población más afectada por la PE procede de hogares con rentas bajas y con algún miembro desempleado^9^ . Asimismo, la PE se ha asociado a familias monomarentales, jubiladas y a mujeres que se dedican a las tareas domésticas^10 , 11^ .

Intervenciones realizadas en otros países orientadas a aumentar la eficiencia energética en los hogares han mostrado mejorar el confort térmico, aumentar el ahorro y, a nivel de beneficios indirectos, crear una mayor sensación de seguridad y de bienestar^12^ . Asimismo, se ha observado que realizar reformas a nivel arquitectónico puede llegar a reducir significativamente la mortalidad invernal^7^ . Sin embargo, las evaluaciones de intervenciones sobre la pobreza energética son escasas. Por ello, el objetivo del estudio fue conocer las valoraciones de una intervención orientada a reducir la PE, incoporando tanto la perspectiva de las personas destinatarias, como de las personas trabajadoras implicadas en el programa.

## MÉTODOS

Se realizó un estudio piloto cualitativo, descriptivo y exploratorio, desde la perspectiva fenomenológica^13^ . Esta investigación forma parte de la evaluación del programa “Energía, la justa”, impulsado por el Ayuntamiento de Barcelona durante el primer semestre de 2016^14^ . El objetivo de este programa fue reducir la PE en poblaciones vulnerables mediante una intervención en tres ámbitos. Primero, promoviendo hábitos de eficiencia energética para lograr ahorro; segundo, optimizando los servicios energéticos como el descuento en las facturas a través de la tramitación del bono social; y tercero, instalando medidas de microeficiencia como las bombillas de bajo consumo. Por otra parte, el programa incluyó un eje de inserción laboral para personas desempleadas de larga duración, a las cuales se les ofreció formación para ser “agentes energéticos” (AE) y realizar la intervención en los hogares. El estudio piloto se realizó entre octubre de 2016 y marzo de 2017.

La evaluación cualitativa se realizó mediante 12 entrevistas semi-estructuradas, a ocho mujeres y cuatro hombres. La muestra estuvo compuesta por los siguientes agentes claves: tres personas destinatarias del programa, tres AE y seis personas que trabajaron en la coordinación del programa, entre las que se incluyeron a una técnica de territorio que realizó tareas de enlace entre los agentes del territorio, además de seleccionar a los AE; un coordinador técnico, responsable de la formación y el asesoramiento a los AE; una coordinadora de programa, responsable de las entidades del territorio; y tres trabajadoras sociales de servicios sociales que realizaron el contacto con las familias candidatas a participar.

Se diseñó un guión de entrevistas en base a la valoración de los agentes clave sobre la puesta en marcha y coordinación del programa, la implementación e impacto de la intervención ( Cuadro 1 ). La selección de los(as) entrevistados(as) se realizó mediante un muestreo teórico, estratificado por subgrupos. El estudio contó con la aprobación del comité ético del Hospital del Mar de Barcelona (con código 2017/7286/I) y todas las personas informantes firmaron un consentimiento informado.

**Cuadro 1:** Resumen del guión de entrevista estructurada y código de participantes de los agentes clave.

Agentes clave	Temas entrevista semi-estructurada
Personas destinatarias (P6, P7, P9)	Valoración del contacto con las personas destinatarias y sobre la posibilidad de participación (Ej. ¿Cómo le informaron sobre la posibilidad de participar en este programa? En general, ¿cómo valora estos primeros momentos?) Valoración general de la implementación del programa, las visitas domiciliarias y las actuaciones de los profesionales a cargo (ej. ¿el programa se ha desarrollado tal y como le habían comentado? ¿Qué es lo que más le ha gustado y lo que menos? ¿Se le han recomendado cambios estructurales en su hogar? ¿Cuáles? ¿Los ha podido introducir? Valoración de este aspecto) Valoración del impacto del programa, valoración positiva o negativa, sugerencias y comentarios (¿Cree que esta intervención ha mejorado de alguna manera su salud? ¿Le ha ayudado a mejorar su bienestar y confort en el hogar? ¿Qué impacto ha percibido en el rendimiento energético – aprovechamiento del calor, ahorro…?)
Agentes energéticos (P1, P3, P8)	Valoración sobre la coordinación y la participación (Ej. ¿Cómo cree que es la coordinación entre los diferentes profesionales e instituciones involucradas en el programa? ¿Cree que llega a las personas que más lo necesitan? ¿Cómo se podría mejorar el diagnóstico de posibles destinatarios(as)?)
Coordinadores de programa (P2, P4, P5, P10, P11, P12)	Valoración general de la implementación del programa (Ej. ¿Cuáles son los principales aspectos positivos del programa? A la hora de implementar el programa ¿cuáles son los principales facilitadores y barreras?) Valoración sobre los potenciales efectos del programa (Ej. ¿Qué beneficios cree que aporta este programa a sus destinatarios(as)? ¿Puede tener también efectos negativos sobre los destinatarios(as)?)

P: persona entrevistada

Las entrevistas fueron grabadas en audio y, una vez transcritas, se realizó un análisis de contenido temático utilizando el *software* Atlas-ti. Se identificaron los ámbitos más relevantes según la perspectiva de los agentes clave. Una vez identificadas las categorías, se examinaron mediante un proceso de comparación con el resto de los datos^15^ . Como criterio de cierre se utilizó la saturación del discurso. Los resultados fueron triangulados por diferentes personas investigadoras que participaron en el estudio.

## RESULTADOS

Los resultados se presentan según la perspectiva de los diferentes agentes claves.

### Confianza en el Contacto Inicial y Desconfianza en Mostrar las Condiciones de la Vivienda

Con respecto a la puesta en marcha y coordinación de la intervención ( Cuadro 2 ), las personas destinatarias vieron positivo que el contacto inicial viniese de la trabajadora social, considerada de confianza. Se valoró la claridad de la información recibida por los AE, como también el buen trato y la buena disposición para colaborar en caso de que hubiera algún problema o urgencia. De hecho, el vínculo creado con los AE fue un facilitador para que se llevara a cabo el programa.

**Cuadro 2:** Valoración de la puesta en marcha y la coordinación de la intervención sobre pobreza energética según diferentes agentes clave.

Agentes claves	Sub-categorías	Verbatims
Personas usuarias	Confianza en persona de contacto	P9: “Bueno, me ofreció la confianza de estar en las oficinas de un estamento oficial, como es Habitatge”
	Buena disposición de los agentes energéticos bien valorada	P7: “Sí, aparte te traían hojas explicándotelo, por si tenías alguna duda…, y bueno, el teléfono de contacto para cualquier cosa, que los llamara, que si venían para cortarte el subministro o algo,…, que llamase urgentemente…” P6: “Tanto el trato, la forma de hablar, sin prisas ... les pongo un 10”
Agente energético	Frustración por falta de recursos	P1: “En el material ha habido muchísima descoordinación, al menos donde yo estaba, ha faltado bombillas y te sabe muy mal. Porque la persona que habla con el usuario le sabe fatal decirle “bombillas no tengo”
	Desconfianza en determinadas poblaciones con vulnerabilidad	P3: “… también hay mucha gente escarmentada, que los han jodido las eléctricas unos palos, que claro. Nosotros íbamos con el chaleco, con la identificación del Ayuntamiento,... pero, aun así, había…recelos.” P1: “…barreras son la gente que lo tiene todo pinchado y no te dejan entrar en su casa porque se creen que los vas a denunciar”
	Desigualdad de género e ilegalidad en alquiler en inmigrantes	P3:”Gente musulmana, que si no estaba el marido, “yo no firmo nada, yo tal...”, “bueno pues cuando esté su marido me llama otra vez y yo vuelvo...”… a veces hay mucha gente que vive aquí y es que no habla nada. P1:”… sobre todo la inmigración, que los pisos no son suyos, que se los ha alquilado otra persona, y que tienen miedo que entre a su casa y que denuncien al que le ha alquilado el piso porque es realquilado.
Coordinadores	Motivación e implicación fueron claves	P2: “...yo creo que, más o menos, todos lo que procuramos fue dar desde el inicio,…, en el sentido de ‘tenemos este reto delante y lo podemos lograr... entre todos, conseguimos creérnoslo entre todos...también, lo hacía todo muy nuevo, estábamos todos allá para salirnos, íbamos echando y, al final ha salido”
	Dificultad de contactar con poblaciones en situación ilegal	P5: “…gente que tiene los suministros de forma ilegal, porque necesitaba luz o gas o agua..., porque en su día no tenía recursos, no recursos económicos, sino recursos para solucionar, gente desamparada que no sabía, porque Servicios Sociales, por colapso, falta de medios, no podía solucionarle la situación,…, entonces, esta gente, a menudo era reacia a que entráramos a su casa”
	Diferencia de género en la selección de personal limitó la igualdad	P4: “…creo también que otro aspecto negativo es… es decir, se escogió un perfil de la construcción cuando creo que se ha visto luego que no ha sido necesario. Creo que eso también limitó mucho en cuanto a temas de igualdad, porque claro, el porcentaje de hombres respecto al porcentaje de mujeres, dentro del proyecto, era muchísimo más elevado, más o menos, 70 hombre por 30 mujeres”

P: persona entrevistada

Según los AE, el hecho de no disponer siempre de los materiales necesarios para hacer la intervención generó sentimientos de frustración. Por otra parte, entre las barreras de contacto, hubo gente que desconfió del programa, bien por haber tenido malas experiencias previas con las compañías energéticas o porque obtenían los suministros de energía de forma ilegal. En la población inmigrante, hubo casos con barreras idiomáticas, como las mujeres que no hablaban castellano y que, además, dependían del consentimiento del marido para poder participar. También se comentó que una parte de la población tenía miedo a participar, sobre todo inmigrantes que subalquilaban habitaciones.

Según las personas coordinadoras y los AE, la motivación y la implicación fueron factores claves, ya que, si bien a veces se tuvo que improvisar, la convicción por parte de los agentes facilitó su desarrollo. También, comentaron que el programa no logró contactar con personas que obtenían sus suministros de energía de forma ilegal o no tenían relación con los servicios sociales del barrio. Por otra parte, se señaló que hubo una clara desigualdad de género en la selección del personal de los AE, con un predominio masculino asociado al perfil laboral de la construcción.

### La Empatía como Factor Facilitador y los Problemas Estructurales como Barrera

En cuanto a la implementación de la intervención ( Cuadro 3 ), el acompañamiento que realizaron los AE en los trámites para la optimización tarifaria, las visitas domiciliarias y la instalación de materiales fueron muy bien valorados por las personas destinatarias. También se valoró la formación en eficiencia energética, aunque un informante comentó que ya conocía estos consejos y que a los AE les faltaba formación.

**Cuadro 3:** Valoración de la implementación de la intervención sobre pobreza energética según diferentes agentes clave.

Agentes claves	Sub-categorías	Verbatims
Personas usuarias	Satisfacción con acompañamiento cambio tarifario	P6: “Ellos consiguen bajar la potencia, según los electrodomésticos y las cosas que tienes en casa, lo que nosotros, particularmente, no nos lo hacen’ [...] ‘...me he sentido muy bien, muy cómoda... en todo momento...Sí, lo han hecho todo ellos”
	Percepción de escasa formación de los agentes energéticos	P9: “…Hubieran necesitado tener un poquitín más de formación y de información, sí, porque incluso yo les dije algunas cosas que ellos no sabían... […]…el último, vino uno de los señores vino solo y el pobre estaba un poco, no avergonzado, pero sí decir, pues es que me falta más información para poderme dar…”
Agente energético	Ayudas beneficiosas pero no resuelven problemas estructurales	P8: “la opción más buena es hacer cambios de materiales, cosas que no podíamos hacer porque no teníamos nosotros y no se podía mejorar mucho, en todo caso, sí que traíamos unos termómetros al menos y les decíamos la humedad y la temperatura adecuada y si tenían estufa, al menos, mirábamos que tuviera la temperatura adecuada y si tenían la tarifa nocturna, la podemos poner por la noche que gastará mucho menos”
	La falta de duración como barrera del programa	P1: “…a última hora ya como no podías gestionar según qué facturas, solamente empoderabas. A mí, eso de castrar así el programa, no lo he visto justo…’ […] ‘una de las cosas negativas de este proyecto es que se ha parado a los 6 meses. Porque cuando una persona le haces un cambio de comercializadora y de tarifa, y se lo haces en el mes 1 o en el mes 2, le da tiempo de visualizar sus facturas si ha mejorado o no.”
Coordinadores	Visitas al domicilio permiten conocer estado de vivienda	P10: “Bien, porque se trabaja, “in situ”, en el propio domicilio de la persona, donde se puede ver y detectar el estado y las condiciones y después de que ya directamente hay una relación con los usuarios y que se los explica más en detalle…”
	Experiencia previa de AE facilitó la empatía	P2: “Los agentes e informantes energéticos son conocedores de primera mano, en muchas ocasiones, de las situaciones de pobreza energética, en este sentido, se considera que este hecho puede facilitar la comunicación con los ciudadanos y ciudadanas atendidos.”
	Burocracia en los trámites fue una barrera	P4: “…una barrera pero de ‘ahora te pido este papel, ahora te pido el otro, y ahora, de repente, lo que se hacía aquí ahora ya no se hace aquí, ahora sólo se puede hacer por teléfono…’. Y marear y dar muchas vueltas… Entonces, esto, también ha sido una barrera bastante importante, que no han facilitado nada los trámites”

Según los AE, se lograron implementar acciones que favorecieron el ahorro y mejoras en la vivienda. No obstante, se comentó que en los pisos con problemas estructurales, como en el caso de filtraciones de aire por ventanas o puertas, el programa no logró obtener mejoras al no contar con los recursos necesarios para realizar reformas. Con respecto a la duración del programa, se consideró que fue demasiado corto, con lo cual hubo intervenciones que no llegaron a finalizarse. Además, la falta de tiempo limitó realizar un seguimiento.

Según las personas coordinadoras, las visitas domiciliarias sirvieron para detectar poblaciones con importantes problemas personales y económicos. La gratuidad de la intervención, y que los cambios en el hogar fueran opcionales, se valoraron como facilitadores para su implementación. Por otra parte, se señaló que el hecho de que los AE hubieran experimentado situaciones de PE facilitó que desarrollaran empatía e implicación en la intervención. Tanto los coordinadores como los AE, identificaron como barrera los trámites que se debían realizar para cambiar las tarifas con las compañías energéticas, considerados demasiado burocráticos y retardando la gestión.

### Empoderamiento, Ahorro y Bienestar

En cuanto al impacto de la intervención ( Cuadro 4 ), las personas destinatarias comentaron que la posibilidad de ahorrar generó mayor sensación de tranquilidad y bienestar. También señalaron que recibir asesoramiento fue percibido como una herramienta de aprendizaje, ya que lograron cambiar hábitos y hacer un mejor uso de los electrodomésticos. Sin embargo, algunas personas señalaron que no creían que la intervención mejorara el confort térmico o el estado de salud.

**Cuadro 4:** Valoración del impacto de la intervención sobre pobreza energética según diferentes agentes clave.

Agentes claves	Sub-categorías	Verbatims
Personas usuarias	Impacto económico y mayor tranquilidad	P7: “En mi caso sí…, poder ahorrarte algo, tal y como está la cosa, y tener tranquilidad… es lo más básico […] lo notas, tanto económicamente como en tranquilidad… claro que lo notas’
	Cambio de hábitos a mayor eficiencia energética	P7: “…aireas toda la casa y… la enciendes para que se caldee. Si no que antes no abríamos… vamos, poco pa’ que no se fuera el calor…’ si antes hacía, por ejemplo, los fines de semana, aquello que pones dos lavadoras... pues ahora voy a probar a poner 3 y claro que ha bajado el consumo, claro que lo notamos…”
	Buena valoración pero sin impacto en mejora térmico	P9: “aparte del asesoramiento, etcétera, de ayudas sociales y demás,… cuando vienes con dolor de espalda, o cualquier cosa, el estar más calentito te ayudará, pues, en este caso te aguantas con el dolorcito. Yo no puedo estar en la temperatura ideal o idónea o mínima que se debería estar, porque no lo puedo pagar.”
Agente energético	El conocimiento contribuyó al empoderamiento	P8: “... sobre todo, los que eran más jóvenes y tenían más empuje que, realmente, les dimos herramientas de empoderamiento. Cómo, veías que decían “teníamos esta opción..., no tenía ni idea, no sé qué, como bajar de potencia, aquí podría ahorrarme mucho dinero...”, y aquí sí que noté que la gente… era consciente de que lo podía hacer”
	No padecer cortes de suministros generó mayor tranquilidad	P1: “sobre todo personas que estaban con avisos de corte de suministro, les produces un año de tranquilidad, de que durante un año no les vayan a cortar…””…le explicas cómo puedes ahorrar con la eficacia energética, pero le explicas también hábitos de consumo…”
	Mayor soporte social	P1: “luego también he visto en personas… que no se sienten solos, que ven que alguien les ha escuchado, que no somos invisibles ante la sociedad…dicen ‘ostras, se han acordado de mí… con lo mal que estoy’”
Coordinadores	Conocimiento bien valorado	P12: “Digamos que aprender, también, a mirar y a leer una factura de la luz, pues también es importante, porque no hay quién lo entienda. Pues esto está bien, yo pienso que esto es una cosa positiva…”
	Impacto potencial en salud mental y otras enfermedades	P2: “Aspectos de salud, aunque no comprobables con un periodo de tiempo tanto corto, existe la posibilidad de experimentar mejoras en términos de salud mental, así como también en casos de enfermedades respiratorias y otros”
	Bajo impacto en cambio de hábitos cuando hay precariedad previa	P11: “... por su propia historia,... son gente que el tema de hábitos los tenían bastante introducidos, eh. Son gente que no gastaba por gastar y que, seguramente, no sabían o no podían cambiar las cosas porque la estructura de la casa no lo permitía, pero no por sus propios hábitos.”

Tanto desde la perspectiva de los AE como de los coordinadores, se comentó que la intervención fomentó el conocimiento sobre un consumo energético más eficiente. Esto se percibió como una estrategia de empoderamiento, sobre todo para los más jóvenes. Según los AE, ofrecer la seguridad de que no habría cortes de suministros disminuyó la preocupación y generó mayor bienestar. Por otra parte, se percibió que los contactos con algunas personas fueron beneficiosos desde el punto de vista del apoyo social, sobre todo en los casos que contaban con pocas redes de apoyo.

Según las personas coordinadoras, la formación de las personas destinatarias contribuyó al ahorro y a experimentar mayor sensación de confort gracias a tener, por ejemplo, la luz encendida durante más tiempo. También, se señaló que el programa tuvo un posible impacto en aspectos de salud mental como el estrés, así como en enfermedades respiratorias. Sin embargo, el impacto fue percibido bajo cuando las condiciones de la vivienda eran extremadamente deficientes. Además, por las mismas circunstancias de restricción económica de algunas familias, el desarrollo de estrategias de ahorro en el consumo de energía se realizaba en algunos casos antes de la intervención.

## DISCUSIÓN

Los principales resultados del estudio señalan que los cambios en las tarifas de los suministros de energía, recibir asesoramiento en materia de eficiencia energética, así como realizar la intervención dentro de los hogares fueron aspectos muy bien valorados. Por un lado, el programa permitió que algunas personas destinatarias disminuyeran los gastos económicos, lo que generó mayor tranquilidad. Por otra parte, el hecho de haber realizado acciones *in situ* , permitió tener un mayor conocimiento sobre las condiciones en las que viven las personas y establecer una relación más próxima con ellas.

En relación a su puesta en marcha, las personas que participaron en el programa valoraron positivamente que fuera una iniciativa municipal y que el profesional de contacto fuera de confianza. Sin embargo, el programa no logró llegar a determinadas poblaciones más vulnerables. Por un lado, aquellas personas que se encontraban en un limbo legal con las compañías de energía mostraron mayor desconfianza a realizar cualquier tipo de intervención en sus hogares. Por otra parte, se comentó que determinados colectivos de inmigrantes subalquilaban la vivienda, una situación de ilegalidad que generó mayor resistencia a participar. Este fenómeno social, más frecuente en el caso de población inmigrante que en el caso de población española, muestra la desigualdad social relacionada con la precarización laboral, la dificultad de firmar contratos con los propietarios de vivienda y la existencia de un mercado de vivienda con elevados precios del alquiler^16^ . Además, se observó un patrón sociocultural relacionado con las desigualdades de género en la medida que las mujeres inmigrantes tienen menos oportunidades de aprender la lengua del país de destino y están más expuestas a sufrir problemas sociales^17^ . Esta situación las posicionó en una mayor desventaja para comprender y participar en este programa, poniendo de relieve la importancia de desarrollar estrategias efectivas para contactar con sub-poblaciones socialmente excluidas y de difícil contacto, sobre todo considerando la feminización de la PE^10^ .

Con respecto a la implementación del programa, hubo una buena percepción sobre el acompañamiento que realizaron los AE para realizar los cambios en el tipo de tarifa contratada, lo que es especialmente relevante si se considera que una de las barreras fueron los trámites burocráticos con las compañías de energía. En este sentido, se ha observado que las facturas de electricidad y gas son percibidas como aquellas que aportan menos información y las menos claras con respecto a los precios de sus servicios, las condiciones de suministro y el tipo de contrato^1^ . Además, la falta de comprensión sobre qué es lo que se paga en un recibo de energía contribuye a la figura del “consumidor vulnerable”, que ve limitada su capacidad de tomar decisiones sobre la gestión de su economía^20^ .

El hecho de que muchos de los propios AE hubieran experimentado PE facilitó la creación de un vínculo con las personas destinatarias. De esta forma, el programa no solo proporcionó apoyo en materia energética, sino que también ofreció apoyo social en la medida que las personas percibieran que una institución valorada como importante se preocupaba por sus condiciones de vida. Existe amplia literatura que muestra el impacto de contar con apoyo social sobre la salud de las personas, lo que se traduce en la necesidad de entender la vulnerabilidad energética como un sistema social complejo que no solo debe considerar el ámbito de la energía^8 , 21 , 22^ .

Para los diferentes agentes clave, el programa tuvo un impacto a nivel económico y en la adquisición de conocimiento sobre derechos, lo que fue considerado una herramienta para el empoderamiento de la población. En general, los informantes destacaron que el ahorro contribuyó a tener mayor sensación de bienestar y menos sentimientos de estrés relacionado con el impago de las facturas. Estos resultados serían consistentes con los encontrados en otro estudio, que mostraba que una intervención que mejora la eficiencia energética en el hogar, genera un impacto positivo al experimentar en las personas usuarias menos dificultades financieras y sentirse más saludables^23^. Sin embargo, en este programa no se observaron mejoras en las personas con viviendas en muy mal estado, ya que requerían reformas de tipo estructural. Todo ello sugiere la necesidad de realizar acciones que intervengan sobre las condiciones sociales y económicas para ayudar a paliar la PE y sus consecuencias.

Entre las limitaciones del estudio destaca el no haber incluido una muestra que pudiera dar cuenta de las experiencias según diferentes características de las personas destinatarias. Sin embargo, el hecho de haber seleccionado a diferentes agentes permitió aportar una visión global del programa. Además, este estudio piloto derivará en otra evaluación cualitativa que permitirá analizar diferentes tipologías de personas destinatarias de esta intervención. En relación a las fortalezas, cabe destacar que este es el primer estudio a nivel del estado español que ha realizado una evaluación cualitativa a diferentes agentes que han participado en un programa en materia de PE. Así, los resultados obtenidos contribuirán a generar evidencia sobre la PE y la salud, un ámbito emergente y muy relevante en nuestro contexto.

En conclusión, el impacto del programa se observó en términos de eficiencia energética, a nivel económico, y también de mejoras en la salud piscosocial. Asimismo, el programa fomentó el empoderamiento en materia energética al aumentar el conocimiento y la capacidad de actuar por los derechos energéticos. Cabe destacar la complejidad que envuelve a la población que vive en situación de PE, teniendo en cuenta tanto los determinantes sociales de la salud, como los diferentes ejes de desigualdad como el género, la diversidad cultural, el estatus migratorio y la clase social, entre otros. Por ello, este tipo de programas deberían desarrollarse en el marco de convenios continuados en el tiempo y asociados a políticas sociales de tipo estructural que incidan sobre los múltiples factores que intervienen en los problemas relacionados con la vivienda.
